# Aged Mitochondrial DNA Is Associated With Aberrant Acute Exercise‐Induced Redox Responses in Human Skeletal Muscle

**DOI:** 10.1111/acel.70678

**Published:** 2026-08-20

**Authors:** Bradley A. Ruple, Nicholas A. Carlini, Jason S. Kofoed, Helya Rostamkhani, Brady E. Hanson, Jesse C. Craig, Shelby C. Osburn, Allison M. Manuel, Paul A. Stewart, Jonathan Wanagat, Ryan M. Broxterman, Joel D. Trinity

**Affiliations:** ^1^ Department of Internal Medicine, Division of Geriatrics University of Utah Salt Lake City Utah USA; ^2^ George E. Wahlen Department VA Medical Center Geriatric Research, Education, and Clinical Center Salt Lake City Utah USA; ^3^ Department of Nutrition and Integrative Physiology University of Utah Salt Lake City Utah USA; ^4^ School of Education and Human Sciences University of Alabama – Birmingham Birmingham Alabama USA; ^5^ Metabolomics Core Research Facility University of Utah Salt Lake City Utah USA; ^6^ Department of Medicine, Division of Geriatrics UCLA Los Angeles California USA; ^7^ Veterans Administration Greater Los Angeles Healthcare System Los Angeles California USA

**Keywords:** aging, exercise, mitochondrial DNA, oxidative stress, redox proteomics

## Abstract

Redox imbalances and mitochondrial dysfunction are key contributors to age‐related declines in skeletal muscle and may contribute to impaired exercise responsiveness. Here, we investigated the influence of aging on skeletal muscle redox at rest and in response to acute exercise, examining how mitochondrial quality and quantity relate to skeletal muscle redox status. Skeletal muscle biopsies were obtained from 12 young (22 ± 4 years) and 10 older adults (66 ± 7 years) before and immediately after 60‐min of high‐intensity knee‐extension exercise. We assessed mitochondrial respiration, mitochondrial DNA (mtDNA) copy number and deletion mutation frequency at baseline, while skeletal muscle redox proteomics was performed on pre‐ and post‐exercise biopsies in a subset of participants. Mitochondrial respiration was preserved with age (max respiration, *p* = 0.123). However, the older adults had a lower mtDNA copy number (*p* = 0.046) and higher mtDNA deletion frequency (*p* = 0.001), with widespread remodeling of the skeletal muscle redox proteome, including altered thiol occupancy of proteins involved in metabolism, immune function, and extracellular matrix organization. In response to exercise, young skeletal muscle exhibited predominantly reversible peptide reductions, whereas preferential oxidation of mitochondrial antioxidant proteins, including PRDX3, occurred in older muscle. Both mtDNA deletion frequency and mitochondrial respiration were strongly associated with exercise‐induced redox modifications in mitochondrial proteins. These findings suggest that aging alters both the regulation and resolution of exercise‐induced redox signaling, with mitochondrial genomic instability and respiration shaping redox responsiveness.

## Graphical abstract

Findings from this study suggest that aging is accompanied by altered skeletal muscle mitochondrial redox regulation and exercise responsiveness, with decreased mtDNA copy number, increased mtDNA mutation deletion frequency, and selective redox remodeling of key mitochondrial proteins representing potential contributors to these changes.

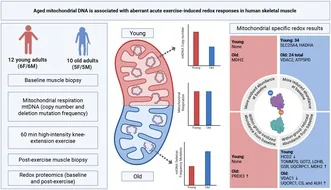

## 1 Introduction

Loss of skeletal muscle mass and function is a defining feature of the aging process and a key determinant of health in older adults (Larsson et al. 2019). While many factors influence this decline, current evidence suggests that mitochondrial dysfunction may play a critical role in age‐related losses in muscle mass and function (Amorim et al. 2022; Joseph et al. 2016; Ruple et al. 2021). In addition to their central role in cellular metabolism and ATP production, mitochondria are responsible for several critical functions, including calcium homeostasis (Pietrangelo et al. 2015), cell signaling (Mammucari and Rizzuto 2010), and immune regulation (Joseph et al. 2016), all of which decline with age. Importantly, mitochondria are also primary regulators of redox homeostasis, due to their role as both a major source and key buffer of reactive oxygen species (ROS). The imbalance between the production and clearance of ROS promotes oxidative stress driving excessive ROS accumulation with aging (Sohal and Orr 2012). This phenomenon has been associated with mitochondrial DNA (mtDNA) mutations and defective removal of damaged/dysfunctional mitochondria, ultimately leading to further impairment of mitochondrial function (Diot et al. 2016; Herbst et al. 2016). Therefore, understanding the cellular modifications governing ROS‐related mitochondrial dysfunction is clinically relevant to minimize age‐related skeletal muscle dysfunction.

Excessive ROS can impair skeletal muscle function through lipid peroxidation, protein oxidation, and DNA damage (Jackson 2009; Powers and Jackson 2008). Proteins are particularly vulnerable to ROS‐mediated modifications, which induce posttranslational modifications (PTMs) in key amino acids such as cysteine, histidine, and methionine (Brandes et al. 2009). Specifically, cysteine residues are uniquely sensitive to redox changes, as the sulfhydryl group is a highly reactive site for allosteric modification (Day et al. 2021). Aging skeletal muscle exhibits an accumulation of oxidative PTMs that can disrupt protein structure, enzymatic activity, and cellular homeostasis (Day et al. 2024; McDonagh, Sakellariou, Smith, et al. 2014). For instance, oxidation of the active site cysteine in protein tyrosine phosphatase 1B reversibly inhibits its phosphatase activity, thereby modulating phosphorylation‐dependent signaling pathways in response to changes in cellular redox state (van Montfort et al. 2003). This observation highlights the need for approaches that can resolve oxidative modifications at the level of individual proteins and residues. Unlike traditional measures of oxidative stress, redox proteomics provides site‐specific resolution of protein modifications induced by ROS. Therefore, this approach allows for the identification of specific skeletal muscle proteins affected by redox imbalance, providing mechanistic insight into how oxidative stress contributes to muscle aging (Abu et al. 2021; McDonagh, Sakellariou, and Jackson 2014).

Physical exercise is a potent stimulus that acutely increases redox stress, where ROS produced during exercise serve as vital signaling molecules (Jackson et al. 2022). The ROS generated from acute exercise can trigger adaptive muscle responses, including enhanced antioxidant defenses and increased mitochondrial biogenesis (Powers et al. 2020). However, few studies have explored the influence of exercise on redox proteomics in skeletal muscle (Kramer et al. 2018; Lamboley et al. 2020; Overmyer et al. 2015; Souza et al. 2018), and to the best of our knowledge, only one study has done so in the context of aging (Pugh et al. 2021). These studies collectively demonstrate that exercise acutely modulates protein oxidation and that mitochondrial proteins are sensitive to oxidative modifications. In the context of aging, Pugh et al. demonstrated that aged individuals had increased oxidative modification in skeletal muscle after acute high‐intensity exercise, indicating diminished redox resilience compared with younger muscle (Pugh et al. 2021). However, the extent to which redox‐sensitive proteins influence mitochondrial respiratory function, and whether mitochondrial characteristics shape these responses, remains unclear.

The purpose of this exploratory study was multifaceted. First, we sought to determine the impact of age on skeletal muscle protein redox levels, mtDNA, and mitochondrial respiration. Second, we aimed to determine whether these baseline mitochondrial characteristics are associated with altered exercise‐induced redox responses. We hypothesized that older adults would exhibit (1) impaired mitochondrial respiration, reduced mtDNA copy number, increased mtDNA deletion mutation frequency, and elevated protein oxidation at baseline and (2) aberrant redox proteomic responses to acute exercise.

## 2 Methods

### 2.1 Ethics Statement

Experimental procedures were reviewed and approved by the University of Utah and the Salt Lake City Veterans Affairs Medical Center Institutional Review Board and were conducted according to the standards set by the latest revisions of the Declaration of Helsinki.

### 2.2 Participants

For both younger and older participants, inclusion criteria required participants to be free of overt cardiovascular and metabolic diseases, with no history of chronic diseases that could impact exercise performance or affect the study results. Inclusion criteria required older participants to be between 55 and 80 years of age and younger participants to be between 18 and 35 years of age. A total of 10 older participants (5F/5M, 66 ± 7 years, 85.3 ± 20.6 kg, 28.4 ± 5.3 kg/m^2^; mean ± SD) and 12 younger participants (6F/6M, 22 ± 4 years, 79.4 ± 14.0 kg, 26.4 ± 4.8 kg/m^2^) provided verbal and written consent before the data collection procedures.

### 2.3 Experimental Design

The study design has been described in detail previously (Ruple et al. 2025). Briefly, prior to the study visit, participants completed a health history questionnaire and a graded single‐leg knee‐extension (KE) exercise protocol to determine their maximal work rate on a KE ergometer. On a separate visit, participants reported to the laboratory following an overnight fast and performed KE exercise starting with a warm‐up at 20% of maximal work rate, increasing by 20% every 3 min until 80% maximal work rate was achieved. Participants then performed a total of nine 3‐min intervals at 80% with 3‐min passive recoveries, for a total of 1‐h from start of the warm‐up to the end of the exercise.

### 2.4 Collection of Skeletal Muscle

Muscle biopsies were obtained before and immediately following the 1‐h KE exercise, from the mid‐belly of the right vastus lateralis. Lidocaine (1%) was injected subcutaneously, superficial to the skeletal muscle fascia. After 10 min of allowing the anesthetic to take effect, a small pilot incision was made using a sterile disposable scalpel, and the 5‐gauge biopsy needle was inserted into the pilot incision until ∼2 cm below the fascia. Approximately 100–150 mg of skeletal muscle was removed with manual suction. Immediately following the biopsy, tissue was rapidly teased of blood and connective tissue, and 30–50 mg of the muscle was stored in ice‐cold media (BIOPS; 2.77 mM CaK_2_EGTA, 7.23 mM K_2_EGTA, 6.56 mM MgCl_2_, 0.5 mM DTT, 50 mM K‐MES, 20 mM imidazole, 20 mM taurine, 5.77 mM Na_2_ATP, and 15 mM phosphocreatine, pH 7.1 at 4°C) for the subsequent respiratory analyses. The remaining tissue was flash frozen in liquid nitrogen and stored at −80°C until further analyses were performed.

### 2.5 Mitochondrial Respiration

In the muscle stored for respiratory analyses, muscle fibers were manually teased apart in 4°C BIOPS by careful use of fine‐tip forceps under a microscope, as described previously (Carlini et al. 2026). The muscle fibers were then permeabilized in BIOPS with saponin (50 μg/mL), with gentle agitation on ice for 30 min. Following permeabilization, muscle fibers were rinsed twice for 10 min in mitochondrial respiration medium (MIR05; 0.5 mM EGTA, 20 mM Taurine, 3 mM MgCl_2_, 110 mM sucrose, 60 mM K‐lactobionate, 10 mM KH_2_PO_4_, 20 mM K‐HEPES, 1 mg/mL BSA, pH 7.1 at 37°C). After rinsing, permeabilized muscle fibers were weighed and placed in an O2k‐chamber containing 2 mL of MIR05, which was continuously stirred and maintained at 37°C. Chamber oxygen concentration was maintained between 250 and 400 μm throughout all experiments.

High‐resolution respirometry (O2k, OROBOROS Instrument, Innsbruck, Austria) was performed in duplicate to measure mitochondrial respiration. The respiration protocol utilized the following sequence of substrates: glutamate (10 mM) and malate (2 mM) for Complex I activity (State 2 respiration), ADP (5 mM) for complex I oxidative phosphorylation (State 3—complex I), succinate (10 mM) to activate Complex II and measure oxidative phosphorylation (State 3—Complex I and II), rotenone (0.5 μM) to inhibit complex I (State 3—Complex II), oligomycin (5 μM) to inhibit complex V, and antimycin A (2.5 μM) to inhibit complex III for background correction.

### 2.6 Mitochondrial DNA Copy Number and Deletion Analysis

Total DNA was isolated from approximately 15–30 mg of skeletal muscle using phenol‐chloroform extraction followed by ethanol precipitation, as previously described (Herbst, Lee, et al. 2021; Herbst, Prior, et al. 2021). DNA concentration and purity were assessed using a Nanodrop spectrophotometer (Thermo Fisher Scientific), Qubit fluorometer (Invitrogen), and Agilent 2200 TapeStation (Agilent Technologies) to ensure sufficient yield, quality, and integrity for downstream applications. The mtDNA copy number and deletion frequency were quantified using droplet digital PCR (ddPCR) on a QX600 Droplet Digital PCR System (Bio‐Rad Laboratories). Two TaqMan‐based ddPCR assays targeting distinct regions of the mitochondrial genome were employed. One assay amplified a stable, non‐deleted region within the mitochondrial minor arc to quantify total mtDNA. The second assay targeted a region within the mitochondrial major arc that is frequently lost in age‐induced deletion mutations to quantify deletion mutation frequency. mtDNA copy number per diploid nucleus were calculated by normalizing mtDNA counts to nuclear DNA (nDNA) using a single‐copy reference nuclear gene. Deletion frequency was calculated as the ratio of deleted mtDNA molecules (detected with in the major arc assay) to total mtDNA copies (amplified in the minor arc assay) (Herbst, Prior, et al. 2021).

### 2.7 Free Radical Measurement

Approximately 10–25 mg of skeletal muscle was used to quantify free radical content with electron paramagnetic resonance (EPR) spectroscopy. Samples were thawed in the superoxide‐sensitive spin probe 1‐hydroxy‐3‐methoxycarbonyl‐2,2,5,5‐tetramethylpyrrolidine (CMH; Enzo Life Sciences, Farmingdale, NY), as described previously. Spectra were obtained with an EMX X micro spectrometer (Bruker, Manning Park, Billerica, MA), and signal area was quantified by double integration of the EPR trace with Xenon software (version 1.1b.51; Bruker) and normalized to tissue weight.

### 2.8 Redox Proteomics

A subset of participants was analyzed for redox proteomics (*n* = 15, 7 young [5F/2M] and 8 aged [4F/4M]). Skeletal muscle was placed in REDOX buffer (50 mM Tris–HCl, 5 mM EDTA, 100 mM NaCl, 1% Igepal‐630, 0.1% SDS) containing 50 mM iodoacetamide (IAA) (57.0215 Da) and degassed with N_2_ and homogenized using tight‐fitting glass pestles and incubated on ice for 1 h before being stored at −80°C. The lysate was acetone precipitated and resuspended in 1× S‐TRAP buffer (50 mM TEAB pH 8.5 and 5% SDS). Total protein content was quantified using the Pierce BCA Protein Assay Kit. 10 μg of protein from each experimental sample was reduced with 10 mM TCEP at 37°C for 30 min, alkylated with 50 mM Iodoacetamide‐d_4_ (CDN Isotopes) (IAAd4 61.041 Da) at room temperature for 45 min in the dark and acidified with 55% phosphoric acid. Each sample was diluted with S‐TRAP binding buffer and loaded directly onto the S‐TRAP micro column, washed 3× with binding buffer, and digested with 0.5 ug of trypsin for 2.5 h at 47°C. The peptides were eluted from the column with 40 μL of 50 mM TEAB pH 8.5 followed by 40 μL of 0.2% formic acid and 40 μL of S‐TRAP elution buffer. The peptides were dried to completion and resuspended in 300 μL of 0.1% TFA. All peptide samples were desalted using Pierce Peptide Desalting Spin Columns (ThermoFisher Scientific) according to the manufacturer's instructions and resuspended in 40 μL of 0.1% formic acid.

### 2.9 Chemicals

LC–MS‐grade acetonitrile and formic acid were obtained from Honeywell Burdick & Jackson, Morristown, NJ, ThermoFisher Scientific, Waltham, MA (Peptide desalting columns), Sigma Aldrich, St. Louis, MO (Tris–HCl, dithiothreitol (DTT), IAA), Promega, Madison, WI (sequencing grade trypsin/LysC), SPEX, Metuchen, NJ (trifluoroacetic acid (TFA)).

### 2.10 MS Analysis

Reversed‐phase nano‐LC–MS/MS was performed on a Bruker nano Elute 2 coupled to a Bruker timsTOF Pro2 mass spectrometer. 150 ng of each sample were injected directly onto the liquid chromatograph reverse‐phase ReproSil C18 150 mm × 0.15 mm nanocolumn (Bruker) heated to 50°C. The peptides were eluted with a gradient of reversed‐phase buffers (Buffer A: 0.1% formic acid in 100% water; Buffer B: 0.1% formic acid in 100% acetonitrile) at a flow rate of 0.5 μL/min. The LC run lasted for 45 min with a starting concentration of 5% buffer B, increasing to 28% buffer B over 40 min, then increasing to 32% buffer B over 2 min, and finally holding at 95% buffer B for 3 min. The separation column was equilibrated with 4 column volumes at 800 bar following each run. The timsTOF Pro2 was operated in PASEF data‐dependent acquisition (DDA) MS/MS scan mode to analyze each experimental sample. The TIMS section was operated with a 120 ms ramp time at a rate of 7.93 Hz and an ion mobility scan range of 0.6–1.4 V·s/cm^2^. MS and MS/MS spectra were recorded from 100 to 1700 *m*/*z*. A polygon filter was applied to select against singly charged ions. The quadrupole isolation width was set to 3 Da.

### 2.11 Data and Statistical Analysis

Peptide abundances were calculated using IonQuant version 1.10.27 in FragPipe version 22.0 software. Statistical data analysis was completed in PyCharm 2022.2.2. An allowance was made for 1 missed cleavage following trypsin/Lys C digestion. The variable modifications of methionine oxidation, N‐terminal acetylation, cysteine iodoacetamide and iodoacetamide‐d4 were considered with a mass tolerance of 15 ppm for precursor ions and a mass tolerance of 10 ppm for fragment ions. A minimum of 1 unique peptide should be reported for all proteins identified.

Baseline statistical analyses were performed using GraphPad Prism (10.6.1). Prior to analysis, assumptions testing for normality were performed using Shapiro–Wilk's test for all baseline dependent variables. For normally distributed data, an unpaired *t*‐test was performed, and for all non‐normally distributed data, a Man–Whitney *t*‐test was performed. Free radical production was the only dependent variable to be analyzed using repeated measures two‐way ANOVA (age × time). Redox proteomics statistical analysis were performed using RStudio (4.5.1). When comparing young and older adults at baseline and post‐exercise, unpaired *t*‐tests were performed. When comparing measurements within the same age group (i.e., young pre vs. young post), paired *t*‐tests were performed. Statistical significance was established using two‐tailed tests with *p* value < 0.05 and a log_2_ fold change (L2FC) greater than ±0.585 (±1.5‐fold change). Significant peptides were submitted to Enrichr for pathway enrichment analysis, with pathway identification performed using the Reactome database (Griss et al. 2020; Kuleshov et al. 2016). To determine whether baseline mitochondrial characteristics influence exercise‐induced mitochondrial redox modifications, we calculated a redox percent change score for each mitochondrial protein, defined as % Oxidized = (Oxidized/(Oxidized + Reduced)) × 100 (Day et al. 2021). Exercise‐induced redox change was defined as the difference between post‐ and pre‐exercise % Oxidized, to account for interindividual variability in baseline redox state. Bivariate correlations were then performed between these redox changes and baseline mitochondrial metrics, with exploratory multiple linear regression with age included as a covariate used to determine whether associations between these were independent of age. Total mitochondrial redox change was also calculated by summing the percentage change across all measured mitochondrial proteins. Proteins were classified as mitochondrial, based on the MitoCarta 3.0 database (Rath et al. 2021). Volcano plots and enrichment graphs were made using RStudio, with all other figures generated in GraphPad Prism.

## 3 Results

### 3.1 Subject Characteristics and Mitochondrial Metrics

**FIGURE 1:**
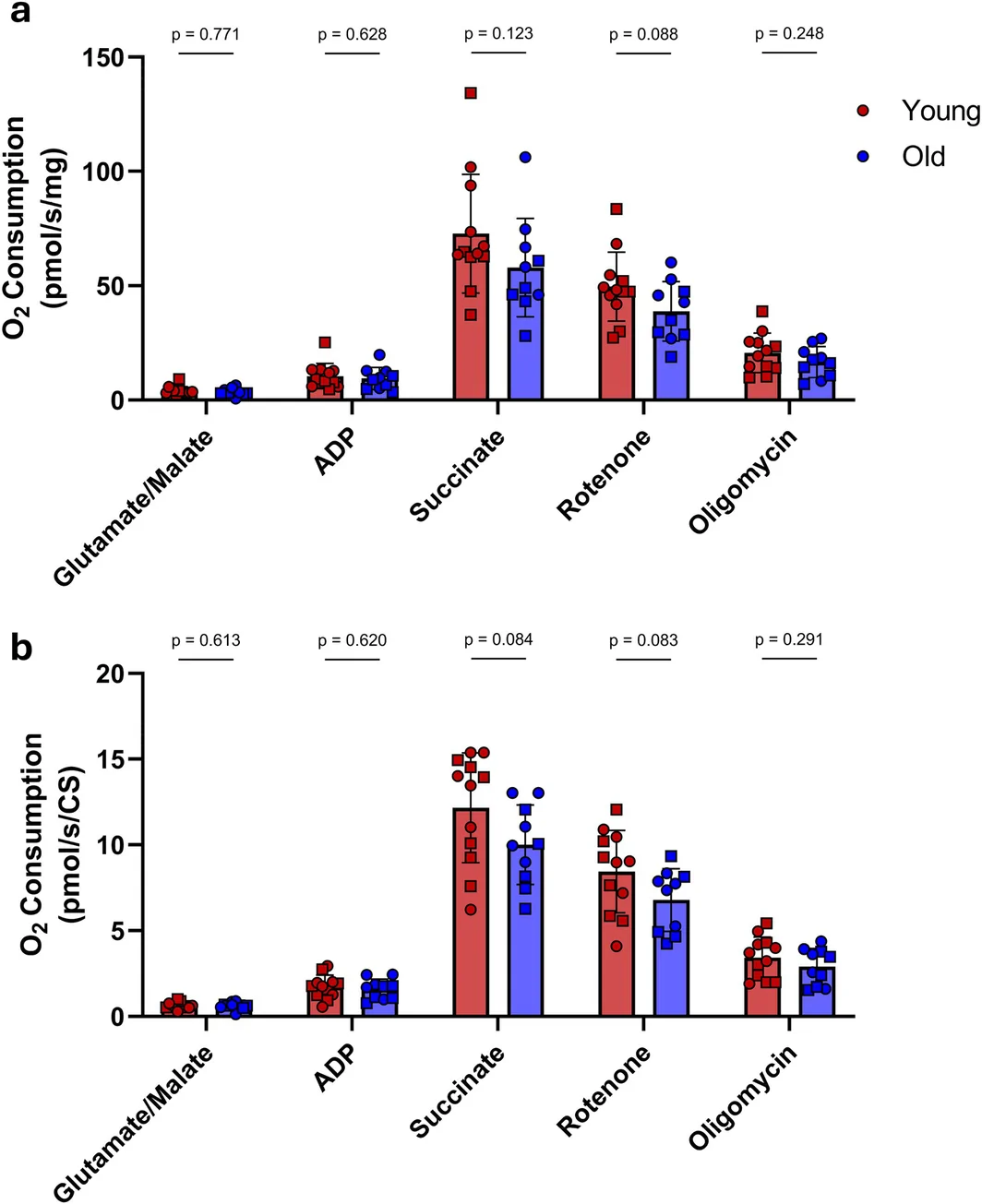
Age‐related differences in skeletal muscle mitochondrial respiration at baseline. Oxygen consumption measured by high‐resolution respirometry in permeabilized vastus lateralis fibers from young (red, *n* = 12) and older (blue, *n* = 10) adults with different substrate conditions. (a) Respiration normalized by tissue weight and (b) citrate synthase (CS). Data are expressed as mean ± SD. Circles denote males and squares denote females.

**FIGURE 2:**
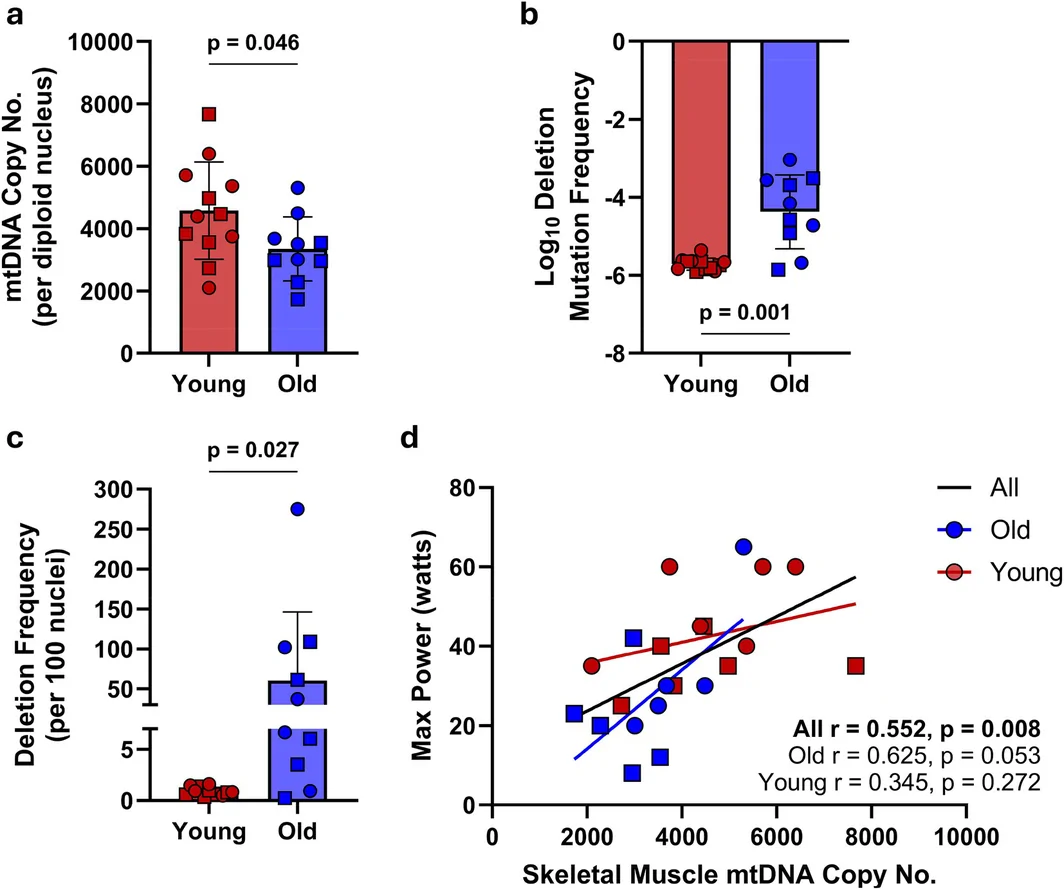
Age‐related differences in mitochondrial DNA copy number, deletion frequency, and functional associations at baseline. (a) Mitochondrial DNA (mtDNA) copy number per diploid nucleus in skeletal muscle from young and older adults. (b) mtDNA deletion mutation frequency. (c) mtDNA deletion mutation frequency normalized per 100 diploid nuclei. (d) Pearson correlations between mtDNA copy number and maximal work rate. Data in panels (a–c) are expressed as mean ± SD. *n* = 12 for young adults, and *n* = 10 for older adults. Across all panels, red indicates young and blue indicates old, while circles denote males and squares denote females.

Young and old were well‐matched for biological sex, body mass, and BMI, with notable differences in age (*p* < 0.001), maximal work rate on the KE ergometer (*p* = 0.022), total cholesterol (*p* = 0.010), and LDL cholesterol (*p* = 0.016) (all, Table 1). In addition, there were no baseline mitochondrial respiration differences (Figure 1a). To account for potential differences in mitochondrial content, mitochondrial respiration was normalized to citrate synthase and age group similarities in respiration remained (Figure 1b). Older adults had a significantly lower mtDNA copy number compared with the younger adults (*p* = 0.046, Figure 2a). The aged group had significantly more mtDNA deletion mutation frequency (*p* = 0.001, Figure 2b), which remained significantly higher after mtDNA deletions were normalized to nuclear number (*p* = 0.003, Figure 2c). Correlational analyses examining relationships between mtDNA markers, baseline participant characteristics, and mitochondrial respiration revealed that, aside from age, the only additional statistically significant correlation was between mtDNA copy number and maximal work rate (Figure 2d).

**TABLE 1:** Subject characteristics.

Variable	Young	Old	*p*
Sex (Male/Female)	6/6	5/5	
Age (years)	22 ± 4 (19–32)	66 ± 7 (57–80)	< 0.001
Body mass (kg)	79.4 ± 14.0 (62.4–104.0)	85.3 ± 20.6 (61.0–126.5)	0.440
BMI (kg/m^2^)	26.4 ± 4.8 (19.7–35.6)	28.4 ± 5.3 (22.4–40.4)	0.361
KE maximal power (Watts)	42.5 ± 12.0 (25–60)	27.5 ± 16.3 (8–65)	0.022
RCR	3.68 ± 0.66 (2.67–4.67)	3.71 ± 1.04 (2.28–5.03)	0.937
Glucose (mg/dL)	86.3 ± 8.2 (73–100)	92.2 ± 10.9 (78–117)	0.181
Total cholesterol (mg/dL)	149.8 ± 17.5 (121–174)	187.9 ± 36.2 (116–232)	0.010
Triglycerides (mg/dL)	73.5 ± 15.9 (44.3–96.8)	92.5 ± 28.6 (49.6–157.0)	0.086
HDL cholesterol (mg/dL)	49.4 ± 6.9 (39.9–60.4)	56.0 ± 11.8 (40.1–74.6)	0.140
LDL cholesterol (mg/dL)	89.2 ± 19.8 (65.0–123.0)	120.3 ± 31.1 (54.5–158.0)	0.016
Total cholesterol/HDL ratio	3.1 ± 0.6 (2.3–4.4)	3.4 ± 0.5 (2.7–4.1)	0.263

*Note:* Data are presented as mean ± standard deviation values, with ranges in parentheses.

Abbreviations: HDL, high‐density lipoprotein; KE, knee‐extension; LDL, low‐density lipoprotein; RCR, respiratory control ratio.

### 3.2 Impact of Age on Protein Abundance

**FIGURE 3:**
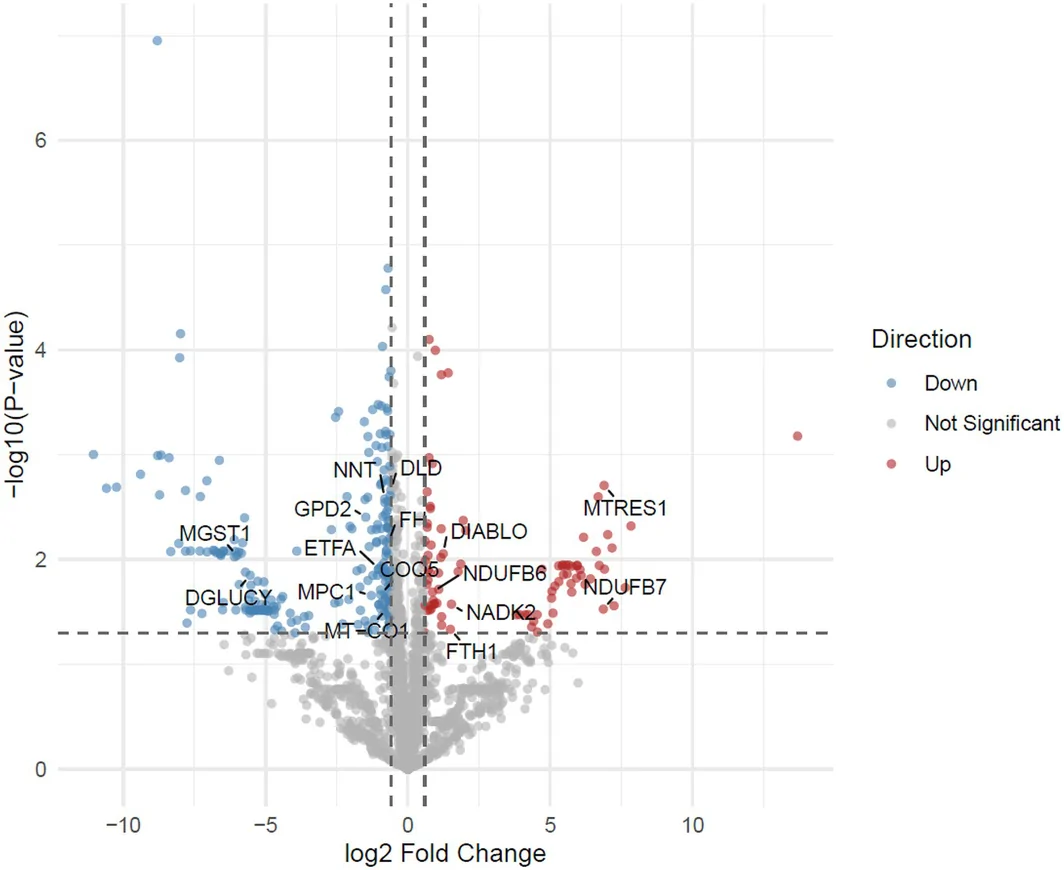
Differentially expressed proteins between young and older adults at baseline. Volcano plot of differential protein expression between young (*n* = 7) and older (*n* = 8) adults at baseline. Vertical dotted lines indicate a log_2_ fold‐change (L2FC) of ±0.58, and the horizontal dotted line indicates a significance threshold of *p* = 0.05 (−log_10_ = 1.3). Proteins exceeding both the fold‐change and significance thresholds were considered differentially expressed. Proteins increased with age are shown in red, decreased with age in blue, and nonsignificant proteins in gray.

To assess whether aging is associated with altered protein abundance relevant to mitochondrial function, protein expression was compared between young and aged skeletal muscle using redox proteomics data (Huang et al. 2023). Relative to young skeletal muscle, 88 proteins were significantly more abundant, and 225 proteins were significantly less abundant in older muscle (Figure 3). Proteins elevated in the older muscle included several mitochondrial proteins, along with proteins involved in redox regulation, iron handling, cellular stress and apoptotic signaling (Table 2). In contrast, proteins that decreased with age included glycolytic enzymes, mitochondrial redox and structural regulators, and key components of excitation–contraction coupling and calcium handling (Table 2).

**TABLE 2:** Age‐associated differences in skeletal muscle protein abundance at baseline.

Gene	Protein	L2FC	*p*
More abundant in old			
NDUFB7	NADH dehydrogenase [ubiquinone] 1 beta subcomplex subunit 7	6.87	0.030
HMOX1	Heme oxygenase 1	6.05	0.012
ABCB7	Iron–sulfur clusters transporter ABCB7, mitochondrial	5.42	0.012
NADK2	NAD kinase 2, mitochondrial	1.53	0.027
DIABLO	Diablo IAP‐binding mitochondrial protein	1.24	0.009
NDUFB6	NADH dehydrogenase [ubiquinone] 1 beta subcomplex subunit 6	0.87	0.020
CTSD	Cathepsin D	0.68	0.002
Less abundant in old			
PHB2	Prohibitin‐2	−6.10	0.007
RYR1	Ryanodine receptor 1	−2.57	0.026
HK1	Hexokinase‐1	−2.44	< 0.001
LDHA	L‐lactate dehydrogenase A chain	−1.07	0.001
PKM	Pyruvate kinase	−0.97	< 0.001
NNT	NAD(P) transhydrogenase, mitochondrial	−0.81	0.002
PFKM	ATP‐dependent 6‐phosphofructokinase, muscle	−0.69	0.028
ATP2A1	Sarcoplasmic/endoplasmic reticulum calcium ATPase 1 (SERCA1)	−0.60	0.025

*Note:* Proteins showing significant differences in abundance between young and old skeletal muscle at baseline. Positive Log2 fold change (L2FC) in redox state indicates a more reduced state in old and negative values indicates a less reduced state in old muscle. L2FC ± 0.58 and *p* < 0.05 considered significant.

### 3.3 Impact of Age on Baseline Protein Redox Status

**FIGURE 4:**
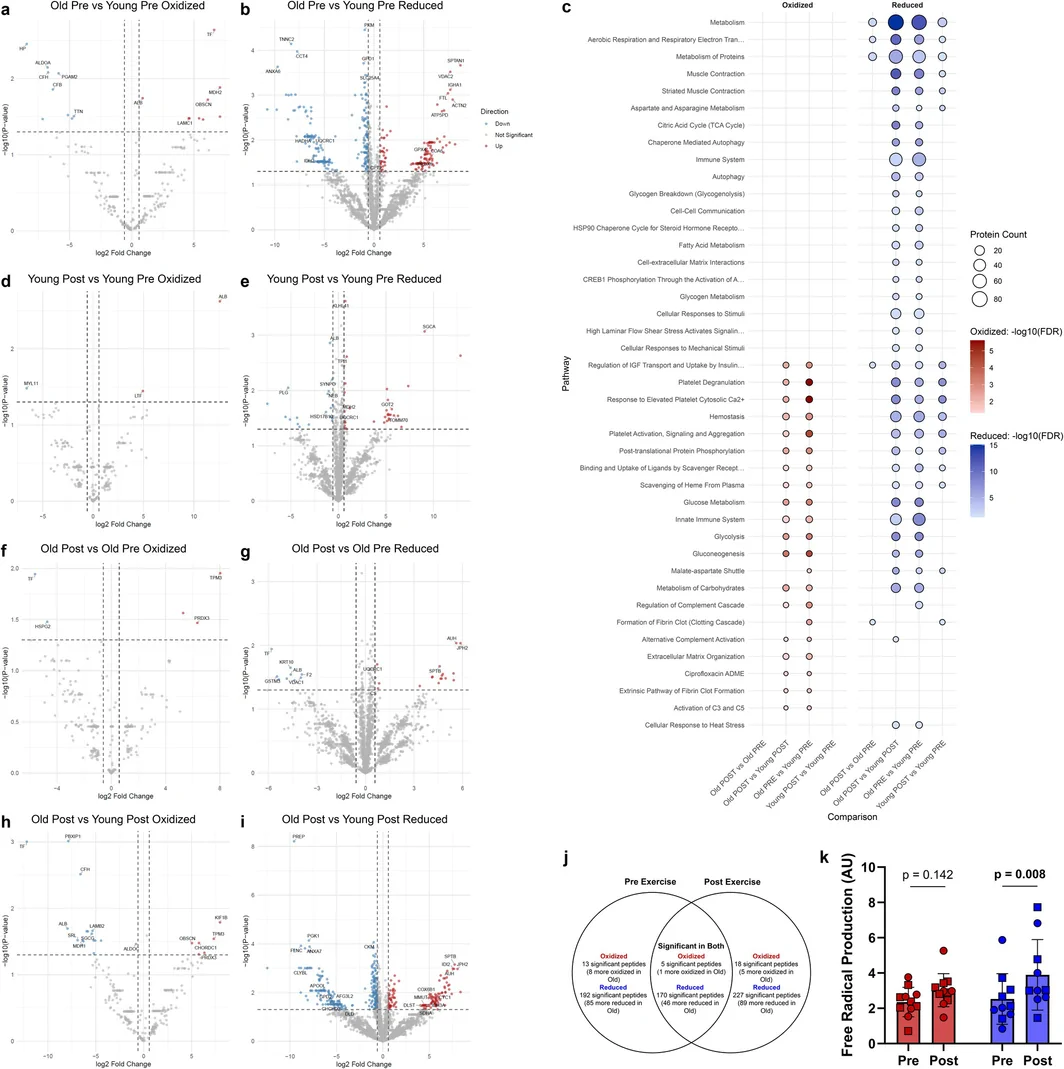
(a–i) Volcano plots of oxidized (a, d, f, h) and reduced (b, e, g, i) peptides from redox proteomics analyses (Old, *n* = 8 and Young, *n* = 7). Comparisons shown are Old Pre vs. Young Pre (a, b), Young Post vs. Young Pre (d, e), Old Post vs. Old Pre (f, g), and Old Post vs. Young Post (h, i). Vertical dashed lines indicate log_2_ fold‐change (L2FC) of ±0.58, and the horizontal dashed line indicates *p* = 0.05 (−log_10_ = 1.3). Proteins exceeding both the fold‐change and significance thresholds were considered differentially expressed. (c) Reactome pathway analysis of significant proteins from all comparisons. Dot size indicates protein count and color indicates significance (−log_10_ FDR). (j) Venn diagram of overlapping and unique significantly altered peptides for age comparisons at Pre‐exercise and Post‐exercise time points. (k) Electron paramagnetic resonance spectroscopy signal as an index of superoxide production before and after exercise. Data are expressed as mean ± SD. Sample sizes were *n* = 11 for young adults (red) and *n* = 10 for older adults (blue). Circles denote males and squares denote females.

A total of 380 peptides exhibited significant differences in redox levels between age groups, corresponding to 264 proteins (Data S1 and S2). Of these, 18 peptides containing oxidized cysteines differed significantly in relative abundance between groups, with nine oxidized peptides showing greater abundance in older adults and nine showing greater abundance in younger adults (Figure 4a). Of the 380 peptides, 362 peptides containing reduced cysteines showed significantly different relative abundance between age groups. A total of 132 reduced peptides showed greater abundance in older adults, while 230 showed greater abundance in younger adults (Figure 4b). The 18 oxidized peptides significantly enriched 40 pathways, and the 362 reduced peptides significantly enriched 136 pathways (Figure 4c). Further, we identified a total of 58 mitochondrial specific peptides that contained reduced cysteines that were significantly different in relative abundance between young and older adults (Data S3). Of note, 34 peptides were more abundant in younger adults, while 24 were more abundant in older adults. In contrast, only a single mitochondrial peptide derived from malate dehydrogenase (MDH) showed a significant difference in oxidation state between age groups, with the oxidized form more abundant in older adults (L2FC = 7.09, *p* = 0.013).

### 3.4 Skeletal Muscle Free Radicals

Skeletal muscle superoxide increased following exercise (main effect of time, *p* < 0.005). Post hoc analysis indicated that this increase was significant in older adults (*p* = 0.008; Figure 4k), although neither the age main effect nor the age × time interaction reached statistical significance.

### 3.5 Exercise‐Induced Changes in Skeletal Muscle Protein Redox Status

Following exercise in young adults, a total of 55 peptides (45 proteins) exhibited significant differences in redox levels. Only three peptides containing oxidized cysteines showed significant changes in relative abundance. Both albumin and lactotransferrin increased the relative abundance of oxidized cysteines following exercise (L2FC = 12.59, *p* = 0.002 and L2FC = 4.95, *p* = 0.036, respectively, Figure 4d), while myosin regulatory light chain 11 (MYL11) decreased the relative abundance of oxidized cysteines (L2FC = −6.60, *p* = 0.033, Figure 4d). Among peptides containing reduced cysteines, 18 peptides were less abundant, while 34 were more abundant following exercise (Figure 4e). The 3 oxidized peptides did not show any significant pathway enrichment, while the 52 reduced peptides significantly enriched 40 pathways (Figure 4c). Regarding mitochondria‐specific peptides, none containing oxidized cysteines exhibited significant changes following exercise; seven peptides containing reduced cysteines differed in relative abundance. Specifically, the peptide 3‐hydroxyacyl‐CoA dehydrogenase type‐2 (HCD2) was less abundant (L2FC = −1.30, *p* = 0.025), while six reduced mitochondrial peptides were more abundant following exercise (Data S3).

Acute exercise significantly altered redox status in a total of 32 peptides (27 proteins) in the older group. Five peptides containing oxidized cysteines exhibited significant differences in relative abundance with three oxidized peptides more abundant and two less abundant following exercise (Figure 4f). Peptides containing reduced cysteines also differed in relative abundance, with nine reduced peptides less abundant and 18 more abundant following exercise (Figure 4g). Reactome pathway analysis indicated that the five oxidized peptides significantly enriched 30 pathways, while the 27 reduced peptides enriched 38 pathways (Figure 4c). When focusing specifically on mitochondrial proteins, only an oxidized peptide derived from thioredoxin‐dependent peroxide reductase (PRDX3) was more abundant following exercise (L2FC = 6.34, *p* = 0.034). In contrast, four mitochondrial peptides containing reduced cysteines exhibited significant changes in relative abundance, with a reduced peptide from voltage‐dependent anion‐selective channel protein 1 (VDAC1) less abundant (L2FC = −4.00, *p* = 0.032), and reduced peptides from cytochrome b–c1 complex subunit 1 (UQCRC1), citrate synthase (CS), and methylglutaconyl‐CoA hydratase (AUH) more abundant following exercise (Figure 4g).

Aging significantly altered post exercise redox levels in skeletal muscle. A total of 420 peptides, corresponding to 285 total proteins, differed with age following exercise. This analysis focused on peptides that were not significantly different between age groups at baseline but exhibited significant differences after exercise (Figure 4j). Exercise altered redox status in 245 unique peptides with 18 peptides containing oxidized cysteines and 227 peptides containing reduced cysteines. The differences reflected that five peptides were more abundant in older adults compared with younger adults following exercise (Figure 4h) and 89 reduced peptides were more abundant in older adults relative to younger adults after exercise (Figure 4i). PRDX3 was the only mitochondrial peptide to have significantly different oxidation following exercise, which is likely due to the increase in oxidation in the old group. There was an identical number of mitochondrial peptides containing reduced cysteines differing between groups, with 21 reduced peptides more abundant in younger adults and 21 more abundant in older adults following exercise (Data S3).

### 3.6 Associations Between Baseline Mitochondrial Metrics and Exercise‐Induced Mitochondrial Redox Changes?

**FIGURE 5:**
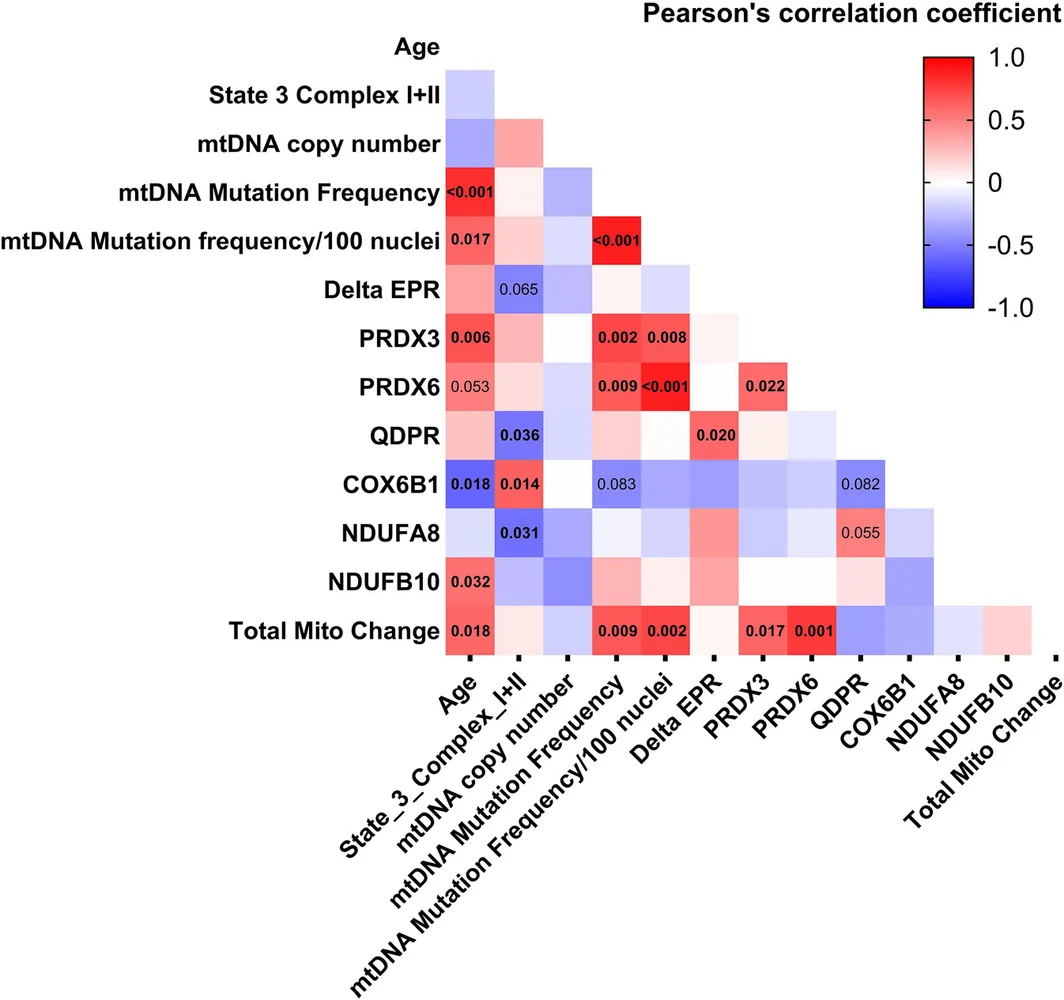
Correlation matrix for baseline mitochondrial characteristics and acute exercise‐induced mitochondrial redox remodeling. Heat map showing Pearson correlations between age, mitochondrial State 3 (Complex I + II) respiration, skeletal muscle mtDNA mutation frequency, mtDNA mutation frequency normalized per 100 diploid nuclei, exercise‐induced change in superoxide, and exercise‐induced redox change scores of select mitochondrial proteins (PRDX3, PRDX6, QDPR, COX6B1, NDUFA8, NDUFB10), as well as total mitochondrial redox change (Old, *n* = 8 and Young, *n* = 7). Redox change scores were calculated as the difference between post‐ and pre‐exercise percent oxidation, where % Oxidized = (Oxidized/(Oxidized + Reduced)) × 100. Total mitochondrial redox change represents the summed redox percent change across all measured mitochondrial proteins. Color indicates correlation direction and magnitude (red, positive and blue, negative), with significant *p* values displayed in bold within cells.

Redox modification of PRDX3 was positively correlated with age (*r* = 0.67, *p* = 0.006), skeletal muscle mtDNA deletion mutation frequency (*r* = 0.72, *p* = 0.002), and deletion mutation frequency per 100 nuclei (*r* = 0.65, *p* = 0.008, Figure 5). Since age was associated with both PRDX3 and mtDNA deletion mutation frequency, an exploratory multiple linear regression analysis was performed to determine whether the association between the two persisted while accounting for age. The relationship between PRDX3 oxidation and mtDNA deletion mutation frequency was no longer statistically significant after adjustment for age (β = 55.46, *p* = 0.151). PRDX6 was similarly correlated with mtDNA deletion mutation frequency (*r* = 0.65, *p* = 0.009), and mtDNA deletion mutation frequency per 100 nuclei (*r* = 0.88, *p* < 0.001). Total mitochondrial redox change was also associated with age (*r* = 0.60, *p* = 0.018), mtDNA deletion mutation frequency per nucleus (*r* = 0.65, *p* = 0.009) and mtDNA deletion mutation frequency per 100 nuclei (*r* = 0.73, *p* = 0.002). The relationship between total mitochondrial redox change and mtDNA deletion mutation frequency was no longer statistically significant after adjustment for age (β = 112.0, *p* = 0.219). Redox status of several proteins was associated with respiratory function (Figure 5). COX6B1 was inversely associated with age (*r* = −0.60, *p* = 0.018) but positively associated with State 3 complex I + II respiration (*r* = 0.62, *p* = 0.014). In contrast, NDUFA8 and QDPR exhibited negative correlations with State 3 complex I + II respiration (*r* = −0.56, *p* = 0.031 and *r* = −0.54, *p* = 0.036, respectively). QDPR also demonstrated a positive relationship with the change in EPR (*r* = 0.59, *p* = 0.020). Lastly, NDUFB10 was negatively associated with age (*r* = −0.55, *p* = 0.032).

## 4 Discussion

Specific redox‐dependent protein alterations that contribute to age‐related losses in skeletal muscle health and mitochondrial quality and quantity remain poorly defined. Herein, we combined redox proteomics with measures of mitochondrial respiration, mtDNA copy number, and mtDNA deletion mutation frequency to examine the impact of age on mitochondrial metrics and their associations with baseline redox status and acute effects of exercise‐induced redox changes. Several key findings were observed with this novel approach. First, older adults exhibit preserved mitochondrial respiration, despite having decreased mtDNA copy number and increased mtDNA deletion frequency. Second, aging was associated with broad remodeling of the skeletal muscle redox proteome at baseline, reflected by shifts in thiol availability and redox occupancy across mitochondrial pathways. This remodeling gave older muscles a more reduced phenotype. Third, acute exercise elicited age‐dependent redox responses, with aging altering both the magnitude and pattern of post‐exercise oxidation and reduction, demonstrating a divergent response to redox handling during exercise‐related stress. Lastly, mtDNA deletion frequency and maximal respiration were associated with targeted redox modifications in mitochondrial and mitochondria‐associated proteins, including PRDX3, PRDX6, and components of the electron transport chain. Collectively, these data suggest that aging is accompanied by altered skeletal muscle mitochondrial redox regulation and exercise responsiveness, with increased mitochondrial genetic instability and selective redox remodeling of key mitochondrial proteins representing potential contributors to these changes.

### 4.1 Aging Alters Mitochondrial DNA Copy Number and Deletion Mutation Frequency

Despite preserved mitochondrial respiration, older adults exhibited significantly lower mtDNA copy number and increased mtDNA deletion frequency, revealing molecular signatures related to mitochondrial aging and a loss of mtDNA integrity, even when oxidative phosphorylation capacity is maintained. Similar to the data herein, mtDNA content and respiratory capacity have been reported to be weakly or inconsistently related (Larsen et al. 2012), supporting the concept that mitochondrial respiration is independent from age‐related reductions in mtDNA copy number and increases in deletion frequency (Sato et al. 2009). However, age‐related changes in mtDNA may increase susceptibility to oxidative stress, aligning with our observation of exercise‐induced increases in skeletal muscle superoxide production, specifically in older adults. We also observed a moderate correlation between mtDNA copy number and maximal knee extension work rate; however, because mtDNA copy number is not a robust predictor of mitochondrial content or respiration, this association should be interpreted cautiously and may reflect indirect relationships with muscle phenotype or metabolic capacity (Larsen et al. 2012). These current findings are consistent with previous evidence of a positive association between mtDNA content and VO_2max_ (Short et al. 2005), alongside an inverse relationship between mtDNA deletion frequency and VO_2max_ (Herbst, Prior, et al. 2021). Together, these findings suggest that aging differentially affects mtDNA and mitochondrial energy production, although these processes remain associated at the physiological level.

### 4.2 Baseline Redox Proteome Reveals Impaired Thiol Cycling With Age

Redox profiling at baseline revealed hundreds of peptides with altered oxidation or reduction status with aging, involving pathways related to metabolism, immune function, transport, and extracellular matrix organization. This is consistent with previously reported large‐scale shifts in thiol occupancy and metabolic protein redox state in aging human skeletal muscle (Lourenco Dos Santos et al. 2015; McDonagh, Sakellariou, Smith, et al. 2014). An increase in the abundance of reduced cysteine‐containing peptides reflects greater availability of free thiols rather than a globally more reduced intracellular environment. Consequently, differences in reduced peptide abundance should be interpreted as changes in cysteine redox occupancy and turnover rather than absolute redox state (Wojdyla and Rogowska‐Wrzesinska 2015). In this context, the greater number of significantly reduced cysteine residues observed in young compared to older adults suggests a greater capacity to maintain or restore cysteine residues to their reduced state. In contrast, the lower abundance of reduced thiols in older adults is consistent with age‐associated impairments in thiol redox cycling, potentially reflecting diminished activity of thiol‐reducing systems or prolonged cysteine oxidation (Droge 2002; Jackson et al. 2022). The impaired thiol cycling may blunt the reversibility of the redox dependent protein regulation, which would limit the ability of skeletal muscle to respond during metabolic challenges. Consistent with this, Reactome pathway analysis of significant redox‐sensitive cysteines and their corresponding proteins revealed enrichment of key mitochondrial pathways, including components of the electron transport chain, tricarboxylic acid cycle enzymes, and mitochondrial redox regulators. In older muscle, altered thiol cycling may also compromise these functions even without a global increase in oxidative damage (Go and Jones 2017). While a clear age‐associated oxidation was not observed in the current study, MDH indicated significantly higher oxidation at rest in the older adults. Despite increased oxidation of MDH with age, there was no difference in mitochondrial respiration between groups. This is likely because MDH is not rate‐limiting for maximal respiratory capacity under saturating substrate conditions (Gnaiger 2020), while subtle redox‐dependent modulations may be masked by enzymatic capacity (Handy and Loscalzo 2012). Therefore, MDH oxidation with aging may reflect altered regulatory or signaling functions rather than overt impairment of mitochondrial respiratory capacity.

### 4.3 Age‐Dependent Redox Responses to Acute Exercise

Acute exercise triggered widespread PTMs of the skeletal muscle redox proteome, with the magnitude and distribution of these changes differing by age. In young participants, exercise primarily altered the reduced peptide pool, with relatively few peptides exhibiting changes in oxidation state. This pattern suggests that exercise‐induced ROS production in young muscle is tightly regulated and rapidly buffered, leading to reversible thiol modifications rather than sustained protein oxidation. This aligns with the role of ROS as important signaling molecules during exercise, where transient oxidation is followed by efficient reductive recovery to support metabolic adaptation (Powers et al. 2016). In contrast, older participants exhibited fewer total redox changes following exercise and a greater proportion of peptide oxidation, including the mitochondrial antioxidant PRDX3. The oxidation of PRDX3 following exercise in older muscle has previously been reported and may further highlight a decreased redox resilience with age (Pugh et al. 2021). These data suggest PRDX3 oxidation as a potential marker of mitochondrial genomic instability, linking accumulated mtDNA damage to altered redox handling during acute stress from exercise. Despite greater oxidation, several mitochondrial metabolic peptides in older adults became more reduced following exercise, indicating that aging does not reflect a uniform loss of redox responsiveness. Rather, aging appears to alter the coordination of redox signaling and recovery, particularly within mitochondria. Collectively, these findings suggest that exercise‐induced redox signaling in young skeletal muscle is characterized by tightly regulated thiol remodeling and efficient recovery, whereas with aging, altered mitochondrial redox regulation may compromise adaptive responses to exercise and mitochondrial resilience. Importantly, while exercise intensity was relative to each participant's maximal capacity, older adults achieved lower maximal work rates. While this is appropriate for comparing physiological response across age, differences in absolute workload may influence the data, with some of the observations reflecting absolute workload rather than aging, per se.

### 4.4 Interplay Between Mitochondrial Function, Genetic Integrity, and Redox Regulation

The exercise‐induced oxidation of PRDX3, together with the observed increase in EPR‐detected superoxide in older adults, is consistent with increased oxidative challenge and altered redox buffering capacity compared with younger muscle. These findings align with previous studies reporting diminished redox resilience in aged skeletal muscle (Jackson and McArdle 2011; McDonagh, Sakellariou, Smith, et al. 2014). PRDX3 oxidation was associated with both age and skeletal muscle mtDNA mutation frequency in unadjusted correlation analyses. However, after accounting for age, the association between PRDX3 oxidation and mtDNA mutation frequency was attenuated and did not remain significant. Suggesting that mitochondrial genetic instability and oxidative modification of redox sensitive proteins may represent age‐associated processes. Recent work has highlighted mitochondrial PRDX3 not only as a scavenger of hydrogen peroxide but also as a redox‐sensitive protein where oxidation reflects failure of mitochondrial antioxidant buffering capacity (Yan and Gan 2023). Hyperoxidized PRDX3 has been proposed as a marker of mitochondrial oxidative stress associated with ferroptotic cell death pathways (Yan and Gan 2023). The strong association between PRDX3 oxidation, age, and mtDNA impairments observed here is consistent with the possibility that mitochondrial genetic instability accompanies a shift from adaptive redox signaling toward greater oxidative modification. In contrast, proteins such as COX6B1, NDUFA8, and QDPR were correlated with mitochondrial respiration and the change in superoxide concentration, suggesting that mitochondrial respiratory capacity may also be associated with redox responsiveness. Together, these relationships support a model in which both mtDNA and respiratory capacity are associated with the oxidative sensitivity of the skeletal muscle proteome with aging. Lastly, these findings identify PRDX3 as a promising candidate marker of altered mitochondrial redox regulation, but do not establish if it directly contributes to age‐related adaptations. Future mechanistic investigation using PRDX3 knockdown or silencing in skeletal muscle coupled with assessments of mitochondrial morphology, respiratory function, and mtDNA will be important to determine how PRDX3 contributes to mitochondrial remodeling with aging and exercise.

## 5 Limitations

This study is not without several noteworthy limitations. First, the relatively small sample size may limit statistical power and generalizability. Second, redox measurements were obtained at a single time point post‐exercise, preventing assessment of oxidation and recovery over time. Analyses were performed on whole‐muscle biopsies, precluding resolution of fiber type specific or submitochondrial redox responses. In addition, the study focused on acute exercise and cannot determine whether the observed redox alterations translate into chronic training adaptations. Similarly, mitochondrial respiration and mtDNA measures were assessed only at baseline and measuring their responses to acute exercise would provide additional insight into how the change in the redox proteome affects mitochondrial function and mtDNA. Furthermore, mitochondrial respiration analyses focused solely on ADP‐supported oxidative phosphorylation and did not evaluate additional aspects of mitochondrial function such as ADP sensitivity, respiratory conductance, or electron transport system capacity using uncoupler titration. Future evaluations of these mitochondrial function related parameters are warranted and may provide novel insight regarding the impact of age on mitochondrial quality. Additionally, mitochondrial structure was also not assessed and combining redox metrics with ultrastructural analyses in future studies could provide further insight into how these molecular changes are affected by mitochondria structure. Lastly, oxidized and reduced proteins were identified using discovery‐based redox proteomics and were not independently validated. Likewise, candidate proteins were identified using unadjusted *p* values, increasing the potential for false positives. Therefore, many markers, like PRDX3, should be considered as promising candidate markers of altered mitochondrial redox regulation with aging and exercise.

## 6 Conclusion

Findings from this study suggest that aging alters skeletal muscle redox regulation at baseline and in response to acute exercise. Aging is associated with widespread remodeling of thiol‐based redox signaling, decreased mtDNA copy number, and increased mutation deletion frequency. Acute exercise revealed age‐dependent differences in redox responsiveness, including selective oxidation of mitochondrial antioxidant proteins such as PRDX3 and increased post‐exercise superoxide production in older adults. Associations between mtDNA copy number and deletion frequency, mitochondrial State 3 respiration, and redox‐sensitive proteins further suggest that mtDNA and functional components are linked to the oxidative sensitivity of the skeletal muscle proteome. Together, these findings suggest that aging is associated with the regulation and resolution of acute exercise‐induced redox signaling, potentially contributing to reduced skeletal muscle adaptability with advancing age.

## Author Contributions

R.M.B. and J.D.T. conceptualization; B.A.R., H.R., J.C.C., S.C.O., A.M.M., P.A.S., J.W., and J.D.T. data curation and analysis; B.A.R., N.A.C., J.S.K., H.R., B.E.H., J.C.C., R.M.B., and J.D.T. performed experiments; B.A.R. and P.A.S. prepared the figures and tables; B.A.R. drafted the original manuscript; B.A.R., N.A.C., J.S.K., H.R., B.E.H., J.C.C., S.C.O., A.M.M., P.A.S., J.W., R.M.B., and J.D.T. edited, revised, and approved the final version of the manuscript.

## Funding

This work was funded by the National Heart, Lung, and Blood Institute at the National Institute of Health (R01HL142603—J.D.T.), the National Institute on Aging at the National Institute of Health (R01AG069924 and P30AG094848‐J.W.) the Veterans Administration Rehabilitation Research and Development Service (I02RX003810—J.D.T. and IK2RX003913—J.C.C.), and Veterans Administration Clinical Science Research and Development Career Development Award (IK2CX002114—R.M.B.).

## Conflicts of Interest

The authors declare no conflicts of interest.

## Supporting information


**Data S1:** Redox mito oxidation supplementary 4.

**Data S2:** Redox mito reduced supplementary 3.

**Data S3:** Redox total oxidation supplementary 2.

## Data Availability

Data that support the findings of this study are available from the corresponding author upon reasonable request.
